# Extracellular Vesicles in Liver Fibrosis: Pathogenic Messengers, Diagnostic Biomarkers, and Therapeutic Nanovectors

**DOI:** 10.3390/pharmaceutics18020230

**Published:** 2026-02-11

**Authors:** Xinyi Zhao, Junyan Zhu, Tianyi Zhang, Wenrong Xu, Hui Qian

**Affiliations:** 1 Key Laboratory of Laboratory Medicine of Jiangsu Province, Department of Laboratory Medicine, School of Medicine, Jiangsu University, 301 Xuefu Road, Zhenjiang 212013, China; 2212313115@stmail.ujs.edu.cn (X.Z.); 2212413064@stmail.ujs.edu.cn (T.Z.); 2 Department of Clinical Laboratory, Shanghai East Hospital, School of Medicine, Tongji University, 150 Ji Mo Road, Shanghai 200120, China; 2112113020@stmail.ujs.edu.cn

**Keywords:** extracellular vesicles, liver fibrosis, pathogenesis, diagnosis, therapy

## Abstract

Liver fibrosis (LF) is the final common pathological outcome of various chronic liver diseases. Advanced LF can progress to severe complications, such as cirrhosis, liver failure, and hepatocellular carcinoma (HCC). Currently, liver transplantation remains the main clinical treatment for advanced LF, but its application is limited by donor availability and unavoidable complications. Extracellular vesicles (EVs), nanoscale particles actively released by hepatic cells, including hepatocytes, hepatic stellate cells (HSCs), and macrophages), circulate in bodily fluids carrying cell-specific cargoes (e.g., RNAs, proteins). EVs mediate intercellular communication via their specific cargo profiles and contribute to the progression in LF. Increasing evidence indicates that tracking changes in the quantity and composition of EVs in LF can aid in disease diagnosis and prognosis prediction. This review discusses the pathological role of EVs in LF development and their potential as biomarkers and therapeutic targets, and provides new perspectives for future research and treatment advances.

## 1. Introduction

Liver fibrosis (LF) is a complex and highly integrated pathological process involving molecular-, cellular-, and tissue-level changes. It is characterized by the excessive accumulation of extracellular matrix (ECM) components, which derives the maintenance of hepatic myofibroblasts and ultimately leads to ECM deposition and organ failure [1]. Various chronic liver diseases such as viral hepatitis, alcoholic liver disease (ALD), metabolic dysfunction-associated steatotic liver disease (MASLD), and autoimmune and genetic disorders, can progress to LF in advanced stages [2].

The progression of fibrosis leads to structural and functional damage to the liver, potentially advancing to cirrhosis and organ failure, which represents a major global health burden. Although fibrosis was once regarded as irreversible, growing evidence indicates that it can resolve in various organs following the removal of the underlying injury. However, this reparative process is often too slow or infrequent to prevent life-threatening complications, especially in advanced fibrosis [3]. Therefore, the development of additional antifibrotic therapies to prevent liver disease progression and HCC is crucial for the treatment of LF. Over the years, numerous in vitro and in vivo models have been developed to address the unmet need for effective and safe anti-fibrotic drugs [4]. Despite advances in understanding the molecular mechanisms of liver fibrogenesis, no approved drug for treating LF is yet available [5]. Thus, identifying more effective antifibrotic targets remains an urgent priority.

Extracellular vesicles (EVs), as defined by the International Society for EVs (ISEV), are lipid bilayer-enclosed particles released from cells that do not replicate independently and exhibit remarkable heterogeneity [6]. This heterogeneity is evident first in their biological origins and physical characteristics. Based on their biogenesis, EVs are broadly classified into two major categories: small EVs (sEVs) and ectosomes [7]. sEVs, which have a diameter of approximately 40–150 nm, are generated via the endosomal system. In contrast, ectosomes originate from the outward budding of the plasma membrane and display a wider size distribution, ranging from 50 to 1000 nm in diameter, including microvesicles, apoptotic bodies, and other larger vesicles [8,9]. Secondly, the proteins present on the surface of EVs are key determinants of their heterogeneity and functional specificity. These surface molecules can be divided into two broad categories. The first comprises “common” or conserved proteins, such as tetraspanins involved in biogenesis, heat shock proteins, and endosomal sorting complex required for transport (ESCRT)-associated proteins, which often serve as universal EVs markers [10]. The second category consists of highly specific “donor cell signatures” or molecular fingerprints. For instance, hepatocyte-derived EVs (hep-EVs) may be enriched with asialoglycoprotein receptor 1 (ASGPR1), whereas EVs derived from immune cell frequently express major histocompatibility complex (MHC) molecules. These donor-specific molecules determine the cellular origin of EVs and mediate their interactions with specific target cells [11]. Furthermore, EVs purification methods, such as ultracentrifugation, size-exclusion chromatography, and polymer-based precipitation, significantly influence the purity of the obtained EV populations. Different methods may enrich EV subpopulations with distinct characteristics and co-isolate contaminants like lipoproteins, thereby adding complexity to EV research [12]. The kinetics of EVs in circulation, including their half-life, tissue distribution, and uptake efficiency of target cells, also vary depending on surface molecules and physical properties, ultimately influencing their biological effects [13]. Different EV subpopulations carry a variety of bioactive cargo such as RNAs, proteins, and lipids, and have been demonstrated to play multifaceted roles in various diseases [14]. Notably, mesenchymal stem cell (MSC)-derived EVs (MSC-EVs) can regulate cell proliferation, apoptosis, angiogenesis, and inflammation, thereby promoting tissue repair and regeneration. Therefore, MSC-EVs have been widely studied and hold considerable therapeutic potential in cardiovascular, neurological, metabolic, and hepatic diseases [15,16]. In this review, we provide an overview of LF and discuss how EVs contribute to its pathologic progression. We also summarize the promising applications of EVs as diagnostic biomarkers and therapeutic agents for LF.

## 2. Development and Pathological of Liver Fibrosis

**Figure 1:**
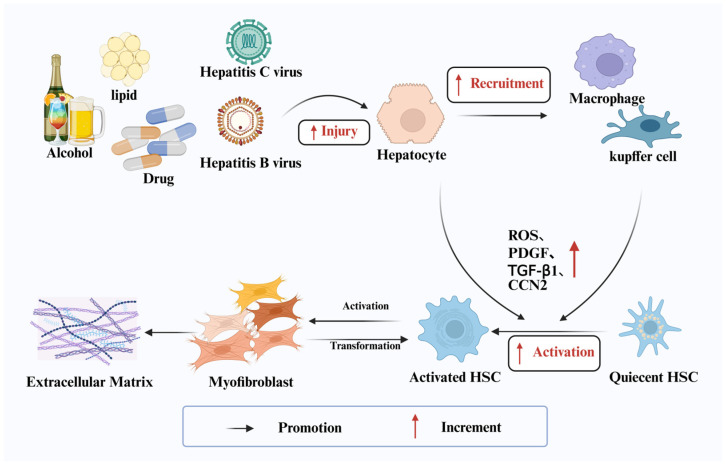
Pathologic mechanisms of LF. Exposure to hepatotoxic agents induces hepatocyte injury, which in turn recruits and aggregates immune cells. Both damaged hepatocytes and inflammatory immune cells release soluble factors and inflammatory cytokines that promote the activation of hepatic stellate cells (HSCs) and their transdifferentiation into pro-fibrotic myofibroblasts. If the underlying cause of liver injury persists, activated HSCs continue to accumulate and produce excessive amounts of extracellular matrix (ECM) proteins, ultimately leading to tissue fibrosis (Created in BioRender. Zhao, X. (2026) https://BioRender.com/1fwp9h6).

LF is an aberrant repair response of the liver to sustained or repetitive injury. Pathologically, it is characterized by excessive production and insufficient degradation of the ECM, leading to the progressive accumulation of scar tissue within the liver. Although this process itself is often clinically silent, its consequences, including disruption of the hepatic architecture, impaired blood flow, ultimate progression to cirrhosis, and portal hypertension, constitute a common final pathway for many chronic liver diseases [17,18] (Figure 1).

Pathophysiologically, the central initiating cells in LF are perivascular hepatic stellate cells (HSCs) [19]. Under conditions of chronic injury and sustained inflammation, these normally quiescent, vitamin A-storing cells become activated and transdifferentiate into contractile and proliferative myofibroblasts [20]. This activation is driven by multiple mediators, such as reactive oxygen species (ROS), platelet-derived growth factor (PDGF), transforming growth factor-β (TGF-β), and connective tissue growth factor (CCN2), released from injured hepatocytes, aggregated Kupffer cells (liver-resident macrophages), platelets, and leukocytes [21]. Activated myofibroblasts produce excessive and aberrant ECM, rich in collagens and glycoproteins. Concurrently, stimulation by factors such as endothelin-1 leads to increased intrahepatic vascular resistance [22]. Fibrous septa form, which connect portal and hepatic venous branches and bypass the hepatocyte plates, leads to hepatocyte ischemia and dysfunction, and progressively leading to portal hypertension. The clinical manifestations of cirrhosis, such as jaundice, variceal hemorrhage, ascites, and hepatic encephalopathy, are precisely caused by this progressive structural and hemodynamic disruption.

The etiology of LF is diverse, primarily including chronic viral hepatitis (hepatitis B and C), MASLD, alcohol-associated liver disease, autoimmune liver diseases, cholestatic liver diseases, as well as certain drug-induced or genetic metabolic disorders [23,24]. While self-limiting acute liver injury usually does not induce significant fibrosis, chronic and repeated injury is key driver of fibrotic progression. Notably, fibrosis at early-stages may be reversible upon removal of the causative insult; however, following sustained chronic injury, the fibrotic process often becomes irreversible [25].

For clinical diagnosis and staging of LF, histopathological assessment via liver biopsy remains the gold standard [26]. However, due to its invasiveness and limitations such as sampling variability, non-invasive diagnostic techniques are increasingly important in clinical practice. Currently, commonly used non-invasive approaches include serum-based models and imaging technologies. Serum-based models assess fibrosis risk by combining routine blood biomarkers with clinical parameters, while imaging techniques such as transient elastography (TE) and magnetic resonance elastography measure liver stiffness to indirectly evaluate fibrosis severity [27,28]. These non-invasive methods are often employed to discriminate between no-to-mild fibrosis and moderate-to-advanced fibrosis, and together with the clinical context, help guide decision regarding liver biopsy.

The cornerstone of current management for LF is etiologic therapy. Effective clearance of chronic hepatitis B or C virus, abstinence from alcohol in patients with alcohol-associated liver disease, weight reduction and control of metabolic abnormalities in patients with MASLD, and relief of biliary obstruction can all halt fibrosis progression and may even promote partial reversal [29]. However, there remains a global lack of direct targeted and widely approved anti-fibrotic drugs. Although numerous pharmacological agents are under investigation for conditions such as non-alcoholic steatohepatitis, their long-term efficacy and safety require further validation [30]. This gap underscores the urgent need to develop novel therapies that specifically target the core mechanisms of fibrosis.

## 3. EVs in the Communication and Diagnosis of Liver Fibrosis

Advanced LF can lead to hepatic cirrhosis and liver failure, with liver transplantation often becoming necessary in the final stages [31]. EVs function as a crucial intercellular communication system, transferring variety of cellular materials and signals such as RNA, DNA, proteins, and lipids, between local or distant cells. This EV-mediated singling plays a key role in the development of conditions such as cancer and metabolic disorders [32,33]. Within the liver, most liver cell types, including hepatocytes, HSCs, cholangiocytes, and macrophages, utilize EVs for intrahepatic communication [34]. Notably, EVs have been shown to participate in the physiopathology of various liver disorders. Depending on their cellular origin, EVs can either promote the formation of LF or inhibit its progression through multiple mechanistic pathways [35]. Beyond their role in intercellular communication, EVs also hold significant promise as diagnostic markers. This potential stems from their cargo such as proteins, mRNAs, and miRNAs derived from their cells of origin, including liver cells. These molecular contents can reflect pathological states, enabling the early detection and prognosis of various diseases, including liver diseases [36].

### 3.1. EVs in Liver Fibrosis

**Figure 2:**
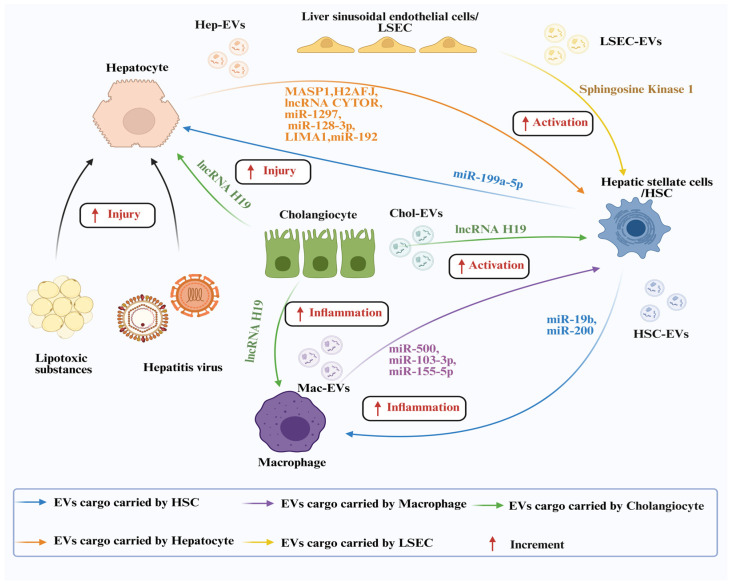
EVs-mediated intercellular communication in the pathology of LF. In the fibrotic liver, hepatocytes, HSCs, macrophages, cholangiocytes, and LSECs communicate through vesicles carrying bioactive molecules and contribute to the disease progression (Created in BioRender. Zhao, X. (2026) https://BioRender.com/7o1h43l).

Long-term insults from alcohol, viruses, and metabolic stress can trigger liver injury, leading to LF, inflammation, and steatosis. Angiogenesis, immunological homeostasis, and ECM buildup are all dysregulated in the development of LF, which eventually results in the advancement of cirrhosis and liver cancer. The progression is determined by complex interactions among key liver cells, including hepatocytes, HSCs, Kupffer cells, liver sinusoidal endothelial cells (LSECs), and immune cells [37]. EVs are critical mediators of this pathological communication, facilitating aberrant crosstalk both within the liver and organ systems. This EV-driven dialog sustains a vicious cycle of inflammation, HSCs activation, and fibrosis (Figure 2) [38].

#### 3.1.1. Hepatocyte-Derived EVs

Hepatocytes, the most prevalent cell type in the liver, are essential for maintaining hepatic homeostasis, and their functional integrity is critical [39]. Hepatocyte damage has been demonstrated to activate HSCs, representing an early event in LF. Hepatocyte nuclear factor 1α(HNF1α), a transcription factor of hepatocyte function, is hypothesized to orchestrate LF progression by modulating crosstalk between hepatocytes and HSCs [40]. This intercellular communication is often mediated by EVs. Various stimuli, such as drugs, viruses, and lipids, can promote EV transmission between hepatocytes and HSCs, suggesting a vesicle-based messaging system that influences LF progression [41]. For Example, hepatocyte-derived EVs (hep-EVs) can deliver specific protein cargoes, such as mannan-conjugated lectin serine protease 1 (MASP1), to HSCs, thereby exacerbating fibrosis [42]. Through transcriptomic analysis, Liu et al. identified the Histone gene *H2AFJ* (H2A Histone Family Member J) and demonstrated that hep-EVs transport *H2AFJ* to activate HSCs via the MAPK/STMN1 (Mitogen-Activated Protein Kinases/Stathmin 1) signaling pathway [43]. Beyond proteins, Hep-EVs also carry pro-fibrotic lncRNAs to HSCs, further promoting fibrosis development [44].

Recent studies indicate that EVs derived from steatotic hepatocytes can promote a pro-fibrotic phenotypic shift in HSCs, implicating them in the pathological communication network described earlier [45,46]. The release of these EVs can be triggered by lipotoxicity. For instance, Hepatocytes exposed to toxic lipids such as palmitate release increased numbers of EVs through death receptor 5 (DR5)-mediated pro-apoptotic signaling [47]. This EV release instigates a pro-inflammatory signal that activates HSCs and accelerates LF. Moreover, Hep-EVs produced from steatosis stimulate HSC activation through various mechanisms. For instance, they may carry specific microRNAs, such as miR-1297, which suppresses PTEN (phosphatase and tensin homolog) and activates the PI3K/AKT (phosphatidylinositol 3-kinase/protein kinase B) pathway. thereby upregulating the expression of fibrogenic genes, such as α-SMA (alpha-smooth muscle actin) and PCNA (proliferating cell nuclear antigen) [41]. Similarly, Steatotic hep-EVs containing miR-128-3p can suppress the expression of peroxisome proliferator-activated receptor γ (PPAR-γ), resulting in the phenotypic change from quiescent to active HSCs [48]. Furthermore, the production of steatotic hep-EVs is associated with upregulation of LIM domain and actin-binding protein 1 (LIMA1). These EVs may increase fibrosis formation by blocking HSC mitochondrial autophagy activation via the PINK1 (PTEN induced putative kinase 1)-Parkin signaling pathway [49]. Chronic viral hepatitis is a leading cause of LF and cirrhosis, with EVs from infected hepatocytes playing a key pathogenic role [50]. Supporting this, EVs extracted from the plasma or serum of hepatitis B virus (HBV)-infected patients have been found to contain HBV DNA and proteins [51]. This suggests that EVs can package viral genetic material, potentially enhancing the susceptibility of recipient cells upon uptake. Furthermore, hepatitis C virus (HCV) infection induces hepatocytes to release EVs carrying specific microRNAs, such as miR-192, which are delivered to HSCs. This transfer promotes HSC transdifferentiation into myofibroblasts via TGF-β1, upregulating fibrotic markers such as α-SMA and COL1A1 (collagen type I alpha 1 chain) [52]. A similar mechanism was reported by Devhare et al., where hepatocyte-derived EVs containing miR-19a suppressed SOCS3 (suppressor of cytokine signaling 3) expression in HSCs. This suppression activated the STAT3 (signal transducer and activator of transcription 3)-mediated TGF-β signaling pathway, thereby driving HSC activation [53]. In summary, substantial evidence confirms that hepatocyte-derived EVs are critical regulators of HSC activation and LF progression.

However, caution is warranted when interpreting findings in this field. Many conclusions are drawn from in vitro models that use specific cell lines or primary hepatocytes under strong stimulatory conditions. These models may overstate the pathological role of EVs and fail to fully replicate the complex in vivo microenvironment. Importantly, as discussed later (Section 4.2), hep-EVs can also exert anti-fibrotic effects under specific conditions, revealing the dual and context-dependent nature of their functions. Such apparent contradictions may arise from the status of donor cells, the heterogeneity of isolated EV subpopulations, or intricate feedback regulatory mechanisms in vivo. Future studies should more precisely delineate the dynamic changes in hep-EV cargo profiles across different pathological stages and under varying stimulatory intensities, and validate these findings using in vivo models to clarify their exact roles within the fibrotic network.

#### 3.1.2. Immune Cell-Derived EVs

Immune cells are vital components of liver, essential for supporting its function and maintaining homeostasis. In response to liver injury, activated HSCs secrete chemokines to recruit immune cells such as neutrophils, macrophages, natural killer (NK) cells, innate lymphocytes, B cells, and T cells [54]. These recruited cells maintain HSC activation through cytokine release or direct contact, driving excessive ECM production and accumulation. Among them, hepatic macrophages have emerged as critical players in the pathophysiology of chronic liver injury and are considered potential therapeutic targets against fibrosis [55]. The hepatic macrophage pool comprises tissue-resident Kupffer cells and monocyte-derived macrophages, both of which interact with HSCs to influence LF [56]. They secrete numerous pro-fibrotic mediators, with TGF-β, being a key cytokine that activates HSCs in paracrine manner [57]. Macrophage-derived EVs have been implicated in this pathological communication [58]. Recent studies demonstrate that macrophage-derived EVs interact with HSCs to modulate LF through specific molecular cargo. For example, Chen et al. showed that EVs from lipopolysaccharide (LPS)-treated macrophages carry elevated levels of miR-500, which promote HSC activation and fibrogenesis by inhibiting mitofusin 2 (MFN2) expression [59]. In a separate study, LPS was found to alter the miRNA profile of macrophage exosomes, specifically enriching miR-103-3p. This EV-borne miR-103-3p targets Krüppel-like factor 4 (KLF4) in HSCs, stimulating their activation and proliferation [60]. Furthermore, a recent independent study reported that co-cultue with LPS-treated macrophages enhanced the migration and proliferation capacity of HSCs, increased oxidative stress, and upregulated fibrosis markers. This effect was attributed to high levels of miR-155-5p in macrophage-derived EVs, which suppressed suppressor of cytokine signaling 1 (SOCS1) in HSCs. The SOCS1 inhibition subsequently promoted the expression of p-Smad2/3, Smad2/3, and RhoA proteins, thereby accelerating fibrogenesis [61].

#### 3.1.3. EVs from Other Sources

Intriguingly, although HSCs are the principal fibrogenic cells in the liver, intercellular communication between HSCs and other cell types remains poorly studied. Nevertheless, it is well established that HSCs play a critical role in the development of LF. In recent years, HSCs-derived EVs (HSCs-EVs) have attracted growing attention. Accumulating evidence indicates that HSCs-EVs contribute to the pathogenesis and progression of liver disease by regulating the metabolism of proteins, lipids, glucose, retinoids, and mitochondria [62]. Previous studies have demonstrated that HSCs-EVs carrying CCN2 or CCN2 mRNA enhance or fine-tune pro-fibrotic signaling [63]. Additionally, HSCs-EVs appear to exert effects on both hepatocytes and HSCs themselves. This was corroborated by Lu et al., who demonstrated that HSCs-EVs accelerate LF progression by delivering miR-199a-5p, which enhances HSC activation and targets Sirtuin 1 (SIRT1) to promote epithelial–mesenchymal transition (EMT) and senescence in hepatocytes [64]. HSCs-EVs can also induce relevant inflammatory phenotypes in immune cells, in addition to their effects on hepatocytes. For instance, EVs enriched with miR19b and miR200, released from activated HSCs, are taken up by macrophage and localize to their membranes. This uptake triggers macrophages to produce and release pro-fibrotic cytokines such as tumor necrosis factor-α (TNFα) and Interleukin-6 (IL-6), thereby accelerating hepatic inflammation and fibrosis [65]. Yana Geng and her colleagues observed a similar outcome in a study where early-activated HSCs-EVs cause Kupffer cells to exhibit a pro-inflammatory phenotype through the Toll-like Receptor 4 (TLR4) signaling pathway [66].

Cholangiocytes are the primary targets in cholestatic liver damage, a condition that significantly contributes to LF and can drive its progression to severe stages. Cholangiocyte-derived EVs have been shown to interact with hepatocytes, macrophages, and HSCs. For example, EVs from cholangiocytes carrying high levels of lncRNA H19 can suppress the expression of small heterodimeric chaperones (SHP) in hepatocytes by inhibiting its promoter activity and mRNA stability. This disruption of bile acid metabolism promotes LF [67]. Futher studies indicate that these EVs can also directly activate and proliferate HSCs, enhancing collagen deposition and accelerating LF [68]. Moreover, lncRNA H19-enriched EVs promote macrophage activation, differentiation, and chemotaxis via the CCL-2/CCR-2 (C-C motif chemokine ligand 2/C-C motif chemokine receptor 2) signaling pathway [69]. Although research on cholangiocyte-derived EVs in LF remains limited, their role is considerable. Future work in this area is expected to clarify the mechanisms of fibrosis in advanced cholestatic liver injury, which may aid in the early diagnosis and treatment of LF.

As the primary cellular component of the sinusoidal wall, LSECs play essential roles in liver homeostasis. Their dysfunction, characterized by loss of fenestration, dedifferentiation, and capillarization, often represents an early pivotal event in LF. Under physiological conditions, LSECs maintain selective filtration, immune regulation, and microenvironmental homeostasis through their fenestrated architecture, and release signaling molecules such as nitric oxide to suppress HSC activation [70,71]. During early liver injury, however, the number and size of LSECs fenestrae diminish or disappear, leading to impaired scavenger function, capillarization, and dysregulated immunomodulation [72]. At this stage, EVs released by activated or dedifferentiated LSECs emerge as important carriers of pathological signals. LSECs-derived EVs (LSECs-EVs) can deliver specific bioactive molecules and directly modulate HSC phenotype through paracrine mechanisms. These EVs not only promote the transdifferentiation of HSCs into activated myofibroblasts, enhancing their migratory and collagen-depositing capability, but may also contribute to sinusoidal capillarization. For example, LSECs-EVs enriched with sphingosine kinase 1 (SK1) can drive the spatial migration of HSCs around sinusoids, thereby promoting fibrotic spread at the tissue level [73]. Moreover, under pathological conditions, elevated serum aldosterone induces excessive autophagy in LSECs, which reduces EV secretion and ultimately facilitates HSC activation and fibrosis progression [74]. Although the specific mechanisms of LSECs-EVs in LF require further elucidation, their role as a critical link between early sinusoidal changes and fibrosis progression is increasingly recognized. They not only reflect LSEC functional transition but also actively regulate HSC activation, immune responses, and vascular remodeling, thereby serving as early responders and bidirectional modulators in LF pathogenesis. Consequently, targeting LSECs-EVs and their cargo may provide novel avenues for early diagnosis and targeted intervention of LF.

Particularly noteworthy is growing understanding that, beyond EVs derived from the aforementioned cell types, EVs-mediated “inter-organ communication” originating from distant organs has emerged as a frontier in LF research, especially in MASLD. On one hand, during intestinal dysbiosis, EVs derived from intestinal cells or gut microbiota can travel via the portal vein to liver. The bacterial components or host-derived mediators they carry directly modulate hepatic immunity and metabolism, establishing a functional dialog along the “gut–liver axis” [75]. On the other hand, in obesity, adipose tissue exists in a state of chronic low-grade inflammation and releases EVs enriched with specific adipokines or miRNAs. Once entering circulation, these EVs are taken up by hepatic cells, where they exacerbate insulin resistance, lipid accumulation, and inflammation, thereby promoting fibrosis progression through the “adipose–liver axis” [76]. These insights redefine LF not merely as a liver-centric condition, but as a systemic disorder orchestrated by multi-organ crosstalk.

However, it should be noted that research on EV mediating “inter-organ communication” remains largely at a phenomenological descriptive stage. While the concepts of the “gut–liver axis” and “adipose–liver axis” are widely acknowledged, several fundamental mechanistic questions remain poorly understood: Which specific cell types within intestinal or adipose tissue release the critical EV subpopulations under disease conditions? What are the precise mechanisms enabling these EVs to cross biological barriers, such as the intestinal mucosa or vascular endothelium? How are their specific cargo recognized and internalized by hepatocytes or non-parenchymal cells in the liver? Moreover, most existing evidence comes from animal models or correlative studies, with a lack of direct causal evidence in humans proving these EVs are necessary pathogenic components [77]. Advancing this frontier will require the use of organ-specific EV secretion-deficient models, sophisticated in vivo tracing techniques, and multi-omics correlation analyses in large clinical cohorts to establish causality and clarify underlying mechanisms.

### 3.2. EVs as Prognostic and Diagnostic Markers for Liver Fibrosis

Liver biopsy has long been considered the gold standard for histologic assessment, diagnosis, and prognosis of LF. However, due to its invasive nature, there is a pressing clinical need to develop non-invasive diagnostic techniques for determining the severity of fibrosis [78]. Current non-invasive approaches to assessing cirrhosis and fibrosis include serum biomarkers, transient elastography (TE), magnetic resonance imaging (MRI), and ultrasound. Nevertheless, these techniques are relatively unreliable for accurately distinguishing between different stages of fibrosis. Moreover, many non-invasive markers may not be able to easily identify the advancement or reversal of fibrosis, as their use in this context has not been widely validated [79]. Hence, new non-invasive diagnostic markers remain to be developed and implemented. All cell types naturally release EVs, which are nanosized particles separated by lipid bilayers and present in all biological fluids [6]. Studies has demonstrated that the pathophysiologic state of the cell of origin influences the composition of EVs [80]. Changes in the quantity, surface markers, and cargo of circulating EVs following liver injury have been identified as potential biomarkers for liquid biopsies of liver disease [81,82]. Interestingly, because EVs carry specific molecular “fingerprints” of their parent cells and their composition changes dynamically with pathophysiological status, they possess the theoretical potential to reflect early abnormal changes in the liver microenvironment before structural damage occurs. This potential has been validated in several experimental studies. For instance, a prospective study in patients with chronic hepatitis C demonstrated that miRNA sequencing of plasma EVs could effectively distinguish significant liver fibrosis based on the expression profiles of EV-encapsulated miR-122-5p and miR-92a-3p. The diagnostic performance of this EV-based miRNA combination surpassed that of some conventional non-invasive serum models [83]. Further research indicated that tracking levels of secreted protein acidic and cysteine-rich (SPARC) protein in HSC-EVs enables non-invasive, dynamic monitoring of the cellular-level efficacy of anti-fibrotic therapies [84]. Collectively, these findings suggest that EVs are not merely passive indicators of pathological states but also active mediators of intercellular communication during early stages of fibrogenesis. Changes in their molecular signatures may help predict subsequent structural remodeling, thereby offering promise for significantly advancing the diagnostic window for liver fibrosis (Table 1).

#### 3.2.1. Protein Cargoes

A small cohort study involving 26 patients with MASLD demonstrated that circulating EVs can predict the severity of LF [85]. In this study, the Kleiner score was used to assess the degree of LF. Joshua A et al. quantified plasma EVs derived from platelets, endothelial cells, and leukocytes, and analyzed EVs surface markers such as CD41a (cluster of differentiation 41a), CD42b, CD31, CD105, CD14, CD16, and CD284 using flow cytometry. Logistic regression analyses were employed to test the independence of association between EV markers and LF after comparing their correlation with conventional LF assessment methods. The results demonstrated that each of these markers enhanced the ability of conventional scoring system to detect LF. In particular, CD14^+^ and CD16^+^ EVs showed stronger potential for predicting the extent of fibrosis. Additionally, glucose transporter protein 1 (GLUT1), carried by hep-EVs, has been identified as a molecular biomarker for early detection of MASLD and a novel non-invasive biomarker for staging LF in MASLD [86]. More recently, proteomic analysis of serum EVs from patients led to the discovery of fibulin-4, a novel protein marker whose level increases with progression of cirrhosis [87]. Interestingly, urine-derived EVs (uEVs) also show promise in identifying LF patients. A proteome analysis of uEVs from 8 patients with ALD-related cirrhosis and 6 healthy individuals revealed differences in uEV concentration, size, and protein composition in cirrhosis patients. This suggests uEVs could serve a new biomarker for ALD-related fibrosis [88]. Due to the ease and non-invasiveness of sample collection, uEVs represent an especially attractive source of biomarkers compared to blood-derived EVs. Therefore, further research into the utility of uEVs in detecting liver disease associated with fibrosis is warranted.

#### 3.2.2. RNA Cargoes

Studies suggest that RNA cargoes, especially miRNAs, play a more significant role than protein cargoes in the diagnosis of liver conditions such as LF. MiRNAs, which are typically about 22 nucleotides in length, are a type of non-coding RNAs essential for the regulation of gene expression. They have been reported to participate in the pathophysiology of many diseases and to regulate key signaling processes such as apoptosis, cell proliferation, inflammatory, and fibrosis [97,98]. miRNAs are present in readily accessible bodily fluids such as blood, urine, and saliva, making them attractive candidates as biomarkers. However, free miRNAs in circulation are susceptible to degradation and lack cellular origin specificity, which limits their utility as standalone diagnostic markers [99]. In contrast, EVs offer a distinct advantage as diagnostic carriers because they can carry and protect nucleic acid molecules, including miRNAs, from nuclease degradation in body fluids, thereby providing more stable and disease-specific molecular signals. In recent years, research on EV-encapsulated RNAs, particularly miRNAs, as diagnostic biomarkers for LF have progressed rapidly. These molecules not only reflect intercellular communication and regulation but also offer, on a technical level, a promising means for non-invasive and dynamic monitoring of fibrosis progression [100]. The combination of EVs and miRNAs essentially forms a “dual-biomarker” system: EVs serve as source-specific carriers, while the miRNA cargo conveys pathological information at the gene-regulatory level. Together, they enhance both the sensitivity and the specificity of diagnosis. Large amounts of miRNAs in EVs can be delivered to recipient cells through intercellular transport, thereby changing the biological phenotype and signaling pathway of target cells. EVs-derived miRNAs from patients have proven valuable as disease biomarkers for a range of conditions [101]. For instance, Li Chen et al. demonstrated that serum EVs from mice with LF exhibited reduced levels of miR-34c, miR-151-3p, miR-483-5p, miR-532-5p, and miR-687 than those from health mice. Administration of EVs from healthy mice to animals with carbon tetrachloride 4 (CCL4)-induced liver injury inhibited hepatocyte damage and attenuated LF. Serum EVs from LF patients and healthy individuals showed the similar phenomenon, with LF patients having lower levels of miR-34c, miR-151-3p, miR-483-5p, and miR-532-5p compared to healthy subjects. Administration of serum EVs from healthy individuals to human-derived HSCs limited fibrosis development by inhibiting HSCs activation [89]. The expression of miR-214 and miR-199a-5p changed in EVs generated by resting and activated HSCs. These miRNAs inhibited HSCs activation by inhibiting CCN2 activity [90,91]. Collectively, EVs from various liver cell types contain a variety of miRNA profiles that contribute to LF and may serve as promising biomarkers for early LF detection [41,48,53,59,60,95,96,102]. These findings underscore the value of EV-encapsulated miRNAs as functional diagnostic biomarkers, as alterations in their expression are not only associated with disease status but also directly participate in the regulation of fibrotic progression. Thus, profiling key miRNA signatures within EVs derived from specific cellular sources can technically enable simultaneous disease identification, staging assessment, and even inference of pathological mechanisms, an outcome difficult to achieve with conventional single-protein biomarkers or imaging techniques [103].

CircRNAs and lncRNAs, which are significant regulators of miRNAs, also undergo specific changes in the pathophysiology of LF in patients with various liver disorders. Serum levels of EVs-carried lncRNA H19 have been found to correlate with the degree of fibrotic liver injury in patients with cholestatic cholangitis and biliary atresia [104]. In a mouse model of arsenite-induced LF, Dai et al. observed elevated levels of lncRNA-MALAT1 (metastasis-associated lung adenocarcinoma transcript 1) in EVs from LF animals compared to controls. Additionally, lncRNA-MALAT1 and micRNA-26b may cooperate to enhance HSCs activation [92]. Another study found that plasma EVs containing circ_0070396 showed higher diagnostic accuracy compared to alpha-fetoprotein (AFP) and were increased in patients with HCC. When combined, AFP and circ_0070396 improved diagnostic performance, outperforming either marker alone in distinguishing HCC from cirrhosis, chronic HBV, and healthy donors [93]. Similarly, Chen et al. demonstrated a marked reduction in circ-0051443 expression in EVs from 60 HCC patients compared to 60 healthy controls, suggesting its potential as a diagnostic indicator [94].

The aforementioned studies across various disorders context further demonstrate the potential of EVs as a non-invasive liquid biopsy platform. Specifically, the analysis of RNAs, especially miRNAs, within EVs is emerging as a promising novel molecular diagnostic technology. This approach integrates the carrier stability and source specificity of EVs with the regulatory significance of miRNAs, enabling sensitive reflection of the dynamic intercellular crosstalk within the hepatic microenvironment. It thus offers the potential to surpass conventional methods in early detection, precise staging, and therapeutic monitoring of liver fibrosis. However, demographic variables such as age, ethnicity, and sex can considerably influence the characteristics of EVs. Consequently, clinical cohort studies should systematically compare EVs from healthy individuals and patients across different stages of LF [105]. Future research must account for these factors to accelerate the discovery and clinical translation of new EV-based biomarkers.

## 4. EV-Based Therapy in Liver Fibrosis

**Figure 3:**
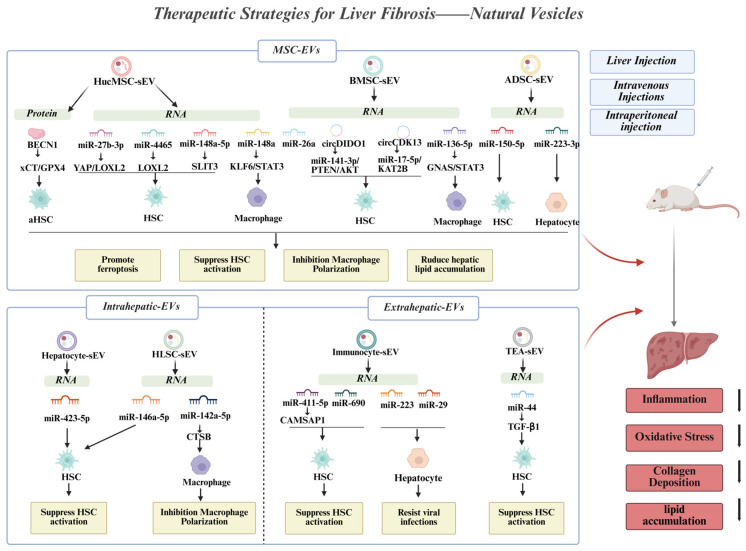
Therapeutic Effects of naturally derived EVs in LF. Following administration Via different routes in rat models, MSC-sEV, Intrahepatic-EVs, and Extrahepatic-EVs carry different cargoes to alleviate the progression of LF by reducing inflammation, oxidative stress, collagen deposition, and lipid accumulation (Created in BioRender. Zhao, X. (2026) https://BioRender.com/xpzmamy).

For many liver diseases, including LF, liver transplantation remains the most effective available treatment. However, its clinical application as a standard therapy is limited by challenges such as donor organ shortages and the necessity for lifelong immunosuppression [106]. Consequently, there is an urgent need to develop novel therapeutic approaches (Table 2). Emerging evidence suggests that EVs derived from a variety of cell types not only play an important role in pathological communication and serve as diagnostic markers but also hold great promise for the treatment of LF (Figure 3).

### 4.1. MSC-Derived EVs

MSC-based therapies represent a promising strategy for treating liver diseases, due to their capacities for self-renewal, differentiation, and paracrine signaling, which collectively promote tissue repair and immunomodulation [107]. Studies has demonstrated that MSC-EVs facilitate intercellular communication and transport paracrine factors involved in angiogenesis, tissue regeneration, and immunomodulation, thereby enhancing the therapeutic efficacy of MSC [108,109].

Current studies have demonstrated that EVs derived from human umbilical cord mesenchymal stem cells (hucMSC-EVs) can alleviate LF [110,111]. One study indicates that LF can be treated using hucMSC-EVs modified with HSCs-targeting peptide [112]. The underlying mechanism involves hucMSC-EVs inhibiting HSC activation, thereby halting the progression of LF [113]. Tan Youwenet al. reported that hucMSC-EVs can induce HSC ferroptosis in human HSC line LX-2 by promoting ROS production and mitochondrial dysfunction. Specifically, hucMSC-EVs regulate the delivery of BECN1 (Beclin-1), which enhances xCT (cystine antiporter light chain)/GPX4 (glutathione peroxidase 4)-mediated ferroptosis in HSCs and ultimately alleviates fibrosis [114]. Moreover, hucMSC-EVs have been shown to attenuate LF by inhibiting the microRNA-148a-5p/SLIT3 (slit guidance ligand 3) axis [115]. Their antioxidant properties also contribute to mitigating liver damage, though the precise mechanism remains unknown [116]. Recently, hucMSC-EVs were found to inhibit helper T cell 17 (TH17) activity and the associated inflammatory milieu, which may halt the progression of LF in primary biliary cholangitis (PBC) [117]. These findings suggest that hucMSC-EVs can modulate the course of LF through immune regulation and other related pathways.

Similar to hucMSC, bone marrow mesenchymal stem cell-derived EVs (BMSC-EVs) also demonstrate effectively and safely therapeutic potential for LF [118]. For example, Rong et al. showed that BMSC-EVs alleviate LF by inhibiting HSC activation through Wnt/β-catenin (wingless/integrated/β-catenin) signaling pathway [119]. Additionally, BMSC-EVs can deliver miR-26a to target the cystine transporter SLC7A11 (solute carrier family 7 member 11), thereby promoting ferroptosis, reducing their activity and slowing the progression of LF [120]. Furthermore, in the context of chronic liver injury, BMSC-EVs can deliver miR-136-5p to macrophages. This attenuates macrophage type 1 phenotype polarization through the GNAS (guanine nucleotide-binding protein)/PI3K/ERK (extracellular signal-regulated kinase)/STAT3 pathway, leading to reduced hepatic inflammation, improved hepatic function, and mitigation of chronic liver disease progression [121]. In another study, Ma et al. established a thioacetamide (TAA)-induced mouse model of hepatic fibrosis and demonstrated that BMSC-EVs could suppress fibrosis by modulating the miR-17-5p/KAT2B (klysine acetyltransferase 2B) axis via circCDK13 [122].

Increasing evidence indicates that therapies based on adipose-derived stromal cells (ADSC) and their secreted EVs (ADSC-EVs) demonstrate significant therapeutic potential in liver injury diseases. This is attributed to their remarkable properties, including tissue repair capacity and immunomodulatory effects, alongside MSC-EVs from bone marrow, umbilical cord, and other sources. Zhang et al. found that ADSC-EVs successfully suppress the production of pro-fibrotic proteins and the EMT in vitro, while also reducing activated HSC proliferation by promoting apoptosis and arresting cells in the G1 phase. Transcriptomic and metabolomic analyses have shown that ADSC-EVs primarily exert their anti-fibrotic effects by inhibiting the PI3K/AKT/mTOR (mechanistic target of rapamycin) signaling pathway and modulating metabolite changes in lipid metabolism [123]. Furthermore, ADSC-EVs can substantially reduce LF by reprogramming glutamine and ammonia metabolism via hepatocyte glutamine synthetase, suggesting a new and promising therapeutic strategy [124]. Additional studies have demonstrated that ADSC-EVs deliver specific cargoes, such as miR-223-3p and miR-150-5p, which contribute to slowing fibrosis progression [125,126].

Furthermore, several studies have shown that MSC-EVs carrying specific miRNAs such as miR-4465 [127], miRNA-34a [128], miR-141-3p [129], miR-148a [130], miR-27b-3p [131], and miR-200a [132] can ameliorate LF by targeting distinct signaling pathway. These involve LOXL2 (lysyl oxidase like 2), Nrf2 (nuclear factor erythroid 2-related factor 2), PTEN/AKT, KLF6 (krüppel-like factor 6)/STAT3, YAP (yes-associated protein)/LOXL2, and ZEB1 (zinc finger E-Box binding homeobox 1)/PIK3R3 (phosphoinositide-3-kinase regulatory subunit 3), respectively.

Despite the extensive research on MSC-EVs summarized above, several significant limitations remain. First, factors such as the MSC source (e.g., umbilical cord, bone marrow, adipose tissue), culture conditions (e.g., serum type, oxygen concentration), and passage number can profoundly influence the yield, composition, and function of the derived EVs, leading to poor reproducibility across studies [133]. Second, most studies utilize total MSC-EVs, which are highly heterogeneous mixtures. The actual therapeutic effects may be attributable to only a specific subpopulation, yet these active components have not been precisely identified. Third, mechanistic explanations often focus on a single miRNA, while potential synergistic or dominant effects of other cargo components are frequently overlooked. More importantly, most animal models are based on acute or subacute injury (e.g., short-term CCl4 injection), which differs substantially from the slow, chronic progression of human liver fibrosis that persists for years or decades. Whether the observed therapeutic efficacy can be sustained under chronic disease conditions remains unclear [134].

### 4.2. Intrahepatic and Extrahepatic-Derived EVs

As the primary functional cells of liver, hepatocytes play a crucial role in maintaining liver homeostasis. While previous reviews have indicated that hep-EVs promote HSC activation and accelerate LF, some studies suggest that they may also exert an inhibitory effect. For instance, Wang et al. assessed hep-EVs in a CCl4-induced mouse model of LF and found that they deliver miR-423-5p to HSCs, suppressing HSC activation and thereby attenuating the development of fibrosis [135]. Another study found that hep-EVs have therapeutic potential for various liver diseases. Similar to the anti-fibrotic properties of MSC-EVs, hep-EVs can reduce HSC activation by inhibiting the TGF-β1/Smad signaling pathway, which subsequently downregulates inflammatory gene expression and cytokine production [136]. Similarly, human liver stem cells (HLSC), an MSC-like population isolated from the liver, exhibit multi-lineage differentiation capacity and immunomodulatory potential, HLSCs have been demonstrated to support liver regeneration [137]. For example, Giulia et al. demonstrated that HLSC derived-EVs (HLSC-EVs) reduce the activated phenotype of HSCs and deliver anti-fibrotic miRNAs [138]. In a mouse model of nonalcoholic steatohepatitis, HLSC-EVs improved liver histology and reduced inflammation and fibrosis by reprogramming hepatic gene expression [139]. Additionally, HLSC-EVs can exert anti-fibrotic effects through modulation of hepatic immune cell. In both in vitro and in vivo studies, Hu et al. found that HLSC-EVs carrying miR-142a-5p attenuated LF progression by targeting cathepsin B (CTSB) and altering macrophage polarization [140].

As noted previously, hepatic immune cells contribute significantly to the progression of LF and represent another key cellular component in the liver. Wang et al. have demonstrated that natural killer cell-derived EVs (NK-EVs) inhibit the activation of TGF-β1-treated LX-2 cells in vitro and prevent CCl4-induced LF in vivo [141]. In a related study, the same authors further elucidated how autophagy inhibition is involved in the anti-fibrotic mechanism of NK-EVs. They found that NK-EVs carry elevated levels of miR-223, and knockdown of miR-223 abolished the ability of NK-EVs to inhibit TGF-β-induced HSC activation. Targetscan analysis identified autophagy-related 7 (ATG7) as a target of miR-223. Moreover, the suppressive effect of NK-EVs on HSC activation was reversed by treatment with the autophagy activator rapamycin or by ATG7 overexpression in LX-2 cells. These findings suggest that miR-223 exerts its anti-fibrotic effect by inhibiting autophagy through the downregulation of ATG7 expression [142].

Beyond their well-characterized functions in mammalian systems, plant-derived EVs from extrahepatic sources have also demonstrated therapeutic efficacy against LF. For instance, Gong et al., reported that tea-derived EVs (TEVs) can inhibit the activation of HSCs. In a CCl4-induced LF model, TEVs treatment significantly improved hepatic histology, inhibited collagen deposition, reduced intrahepatic lipid accumulation, and lowered serum AST and ALT levels. Mechanistically, TEVs carry miR-44, which alleviates fibrosis by blocking TGF-β1 signaling [143]. Additionally, a recent study indicates that EVs derived from cannabis buds may also contribute to LF treatment [144]. Collectively, these findings underscore the broad therapeutic potential of EVs in LF, especially MSC-EVs, which offer a promising, novel approach for future LF treatment and early clinical detection.

### 4.3. Engineered EVs

**Figure 4:**
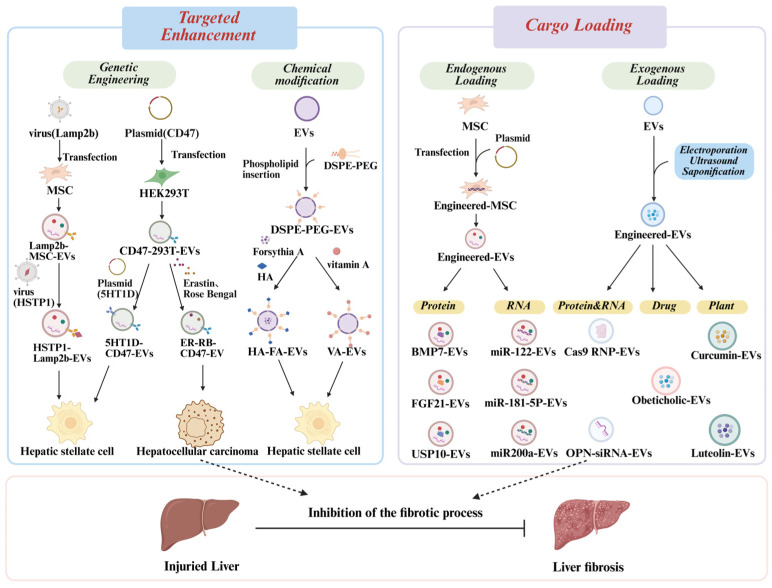
Engineering strategies for EVs. This section outlines two primary approaches for EVs. First, EVs can be genetically engineered and chemically modified at the membrane level to target specific tissue or cell types. Second, therapeutic cargo (e.g., nucleic acids, proteins, or drugs) can be loaded into EVs through endogenous or exogenous methods. Together, these engineering approaches enhance the therapeutic efficacy of natural EVs and provide novel perspectives for treating LF (Created in BioRender. Zhao, X. (2026) https://BioRender.com/ahr18z5).

Building on the evidence summarized above, naturally EVs derived from various sources show considerable promise for treating liver diseases, such as HCC and LF. By facilitating intercellular communication, these EVs can promote liver regeneration and help alleviate oxidative stress, inflammation, fibrosis, and tumor growth. However, the bioactivity of natural EVs is highly dependent on the physiological state of their donor cells, which often lead to inconsistent therapeutic outcomes. Additionally, their clinical utility is frequently limited by poor tissue targeting and nonspecific biodistribution following systemic administration (Figure 4). To overcome these challenges, EVs can be engineered to enhance their therapeutic precision and efficacy. Through the targeted loading of bioactive cargo, including lipids, proteins, miRNAs, drugs, surface modifications that direct them to specific cells or tissues, engineered EVs can achieve superior targeting and functional performance, thereby maximizing their regenerative and therapeutic potential [145]. There are two ways to accomplish these objectives: either directly loading cargo (nucleic acids, proteins, medications, etc.) into EVs using endogenous or exogenous mechanisms, or surface-modifying EVs to target particular tissue or cell types.

Previous studies in various disease models have demonstrated that surface engineering of EVs can enhance their targeting ability and therapeutic efficacy by modifying surface-associated homing peptides or ligands [146]. One common strategy is genetic engineering, in which parental cells are engineered to express specific targeting peptides or proteins, leading to the spontaneous display of these moieties on the membranes of secreted EVs and enabling site-specific delivery [147]. This approach often utilizes lysosome-associated membrane protein 2b (Lamp2b), a widely expressed EV membrane protein. For instance, after screening a phage-display library for peptides that bind activated HSCs, researchers fused the identified peptide HSTP1 (hepatic stellate cell targeting peptide 1) to Lamp2b. In mice with CCL4-induced hepatic fibrosis, the engineered EVs selectively accumulated in HSCs and exerted potent antifibrotic effects [112]. Furthermore, incorporating the “don’t eat me” protein, CD47 on EVs, can help evade phagocytic clearance, prolong circulation half-life, and improve delivery to target sites [148]. This principle has been applied to develop targeted EV platforms. Du et al. loaded CD47 onto EVs via viral transfection and used them to deliver the ferroptosis inducer Erastin and photosensitizer Rose Bengal to HCC tissues, achieving effective tumor suppression through chemiluminescent photodynamic therapy [149]. In another study, Zhang et al. co-labeled EVs with CD47 and antibodies against the 5-HT1D (5-Hydroxytryptamine Receptor 1D) receptor to create a nanoscale delivery system for miR-29b. These engineered EVs selectively reached HSCs while avoiding macrophage uptake, resulting in precise release of miR-29b at fibrotic sites and enhanced antifibrotic activity [150]. In addition to genetic engineering, chemical modification methods enable direct modification of the membrane surface of EVs via lipid insertion, chemical ligation, enzymatic ligation, and affinity binding. A widely used approach employs the FDA (food and drug administration)-recognized DSPE-PEG (1,2-distearoyl-sn-glycero-3-phosphoethanolamine-polyethylene glycol) module, in which the hydrophobic DSPE moiety anchors to the EV membrane, while the hydrophilic PEG spacer is conjugated to a targeting ligand. For example, hyaluronic acid (HA)-modified mammary EVs (mEVs) loaded with forsythin A showed enhanced antifibrotic efficacy by targeting CD44-overexpressing cells [151]. Similarly, vitamin A-conjugated EVs prepared via DSPE-PEG technology effectively delivered cargo to HSCs and attenuated fibrosis in mice [152].

These studies demonstrated that modifying the surface membranes of EVs can enhance their targeting ability and therapeutic potential. Additionally, directly loading therapeutic cargo, such as drugs, proteins, and nucleic acids into EVs can further improve their efficacy against LF. Taking MSC as an example, various biological tools (e.g., plasmids, viral vectors) can be applied to genetically engineer MSCs, whose physiological state directly influences the properties of the EVs they release [153]. This endogenous loading strategy enables MSCs to synthesize specific biomolecules and package them into secreted EVs during biogenesis. Multiple investigations have shown that modifying nucleic acids such as miRNAs within parent cells can significantly affect the therapeutic performance of their EVs. For instance, EVs derived from ADSCs overexpressing miR-181-5p significantly downregulated fibrosis-related markers both in vivo and in vitro, thereby alleviating liver injury and inhibiting the progression of LF [154]. Similarly, EVs engineered to overexpressed miR-200a [132] or miR-122 [155] also demonstrated enhanced anti-fibrotic efficacy to varying degrees. In addition to nucleic acid, endogenous loading of proteins also plays a crucial role in enhancing the therapeutic efficacy of engineered EVs. For example, Zhu et al. utilized virus transfection to genetically modify hucMSC to overexpress bone morphogenetic protein 7 (BMP7), an anti-fibrotic factor. They then used ultracentrifugation to produce engineered MSC-EVs that overexpressed BMP7. Engineered MSC-EVs increased anti-fibrotic activities in vitro and in vivo, diminished HSC proliferation, and accelerated phenotypic reversal of HSCs in comparison to normal EVs [156]. Recent research further indicates that ubiquitin-specific protease 10 (USP10) [157] and fibroblast growth factor 21 (FGF21) [158] are enriched in EVs and contribute to the prevention and treatment of liver disorders, including LF and steatohepatitis.

In addition to the endogenous cargo loading, exogenous techniques such as electroporation, ultrasonication, freeze–thaw cycles, saponin-assisted incubation, and extrusion can be used to directly load therapeutic molecules into isolated EVs. For instance, using an optimized electroporation method, Wan et al. developed genome editing delivery system—EV^RNP^—a novel genome-editing delivery system in which Cas9 ribonucleoprotein (RNP) is loaded into purified EVs. Based on the RNA-guided clustered regularly interspaced short palindromic repeats and associated Cas9 endonuclease (CRISPR-Cas9) platform [159], EV^RNP^ has demonstrated strong therapeutic potential in various liver diseases, including acute liver injury, LF, and HCC, by targeting the PUMA (p53 upregulated modulator of apoptosis), CcnE1 (cyclin E1), and KAT5 (klysine acetyltransferase 5) genes, respectively [160]. Electroporation can also be employed to load siRNA into EVs. In one study, EVs loaded with siRNA targeting osteopontin (OPN), a cytokine involved in oxidative stress and liver fibrosis, more effectively restored liver function than free siRNA-OPN, by suppressing hepatic stellate cell activation and ECM deposition [161]. Interestingly, EVs can also be engineered to carry plant-derived compounds or drugs with known anti-fibrotic activity. For example, luteolin (LUT), a flavonoid with strong antioxidant and anti-inflammatory properties, holds promise for treating liver diseases including LF, but its poor water solubility and rapid metabolism limit clinical application. To overcome this, researchers used ultrasonication to generate LUT-loaded EVs. Owing to their small size, prolonged circulation, sustained release profile, and enhanced tissue penetration, these engineered EVs show remarkable therapeutic potential against LF [162]. Similarly, curcumin [163] and obeticholic acid (OCA) [164] (a drug for treating liver injury) have been successfully loaded into EVs via saponification and ultrasonication, respectively, improving their therapeutic effects. In summary, these engineering strategies help overcome the inherent limitations of natural EVs and provide innovative avenues for managing liver diseases such as LF. EV-based modification technologies represent promising tools for advancing precision and nanomedicine applications.

While engineered EVs undoubtedly hold significant therapeutic potential, their development remains at an early stage and faces several key challenges. First, the efficiency of current engineering strategies is limited: endogenous modifying of parent cells may reduce cell viability or alter normal EV biogenesis, whereas exogenous loading methods such as electroporation or sonication can damage EV membrane integrity and impair the function of native proteins, ultimately affecting their biodistribution and cellular uptake. Second, scaling up production presents practical obstacles, including the stability of genetically engineered cell lines, along with the cost and quality control of large-scale culture and EV purification, all of which must be addressed for successful clinical translation. Third, long-term safety and immunogenicity remain uncertain. Whether overexpressed or externally introduced molecules cause off-target effects or immune responses in vivo, and how engineered EVs are metabolized and cleared in the body, still require systematic investigation. Finally, and most critically, regulatory pathways for EVs remains ambiguous. As complex biological products, EVs currently lack established standards for defining critical quality attributes, such as size, surface markers, cargo loading, purity, potency, sterility, and batch-to-batch consistency, which severely hinders their advancement into clinical trials [165].

**Table 2:** Therapeutic function of EVs in LF.

Source of EVs	Therapeutic Molecules	Signaling Pathway	Animal Models	Method of Administration	Effect of EVs In Vivo	Cell Models	Effect of EVs In Vitro	Ref.
hucMSC	-	-	CCl4-treated mice	IV	alleviate liver damage, reduce collagen deposition	LX-2	inhibit HSCs activation,	[112]
hucMSC	BMP7	-	CCl4-treated mice	IV	improved liver function, reduce collagen deposition	LX-2	inhibit HSCs activation	[156]
hucMSC	BECN1	xCT/GPX4	CCl4-treated mice	IV	reduce collagen deposition	LX-2	promote HSCs ferroptosis	[114]
hucMSC	miR-148a-5p	miR-148a-5p/SLIT3	CCl4-treated mice	IV	improve liver function	LX-2	inhibit HSCs activation	[115]
hucMSC	-	-	CCl4-treated mice	IV	reduce oxidative stress levels, reduce collagen deposition	Hepatocyte	reduce oxidative stress, inhibit cell Apoptosis	[116]
hucMSC	-	-	CCl4-treated mice	Liver Injection	alleviate liver inflammation, reduce collagen deposition, restore serum AST activity	-	-	[110]
hucMSC	-	-	Mdr2−/− mice	IV	improve liver function, alleviate LF	TH17	moderate Th17-induced fibrosis-associated microenvironment	[117]
HucMSC	miR-27b-3p	YAP/LOXL2	CCl4-treated mice	IV	reduce collagen deposition	LX-2	inhibit HSCs activation	[131]
HucMSC	MiR-148a	KLF6/STAT3	CCl4-treated mice	IV	reduce liver inflammation, improve liver function	RAW264.7	modulate macrophage polarization	[130]
HucMSCs	MiR-4465	LOXL2	CCl4-treated mice	IV	reduce collagen deposition	LX-2	inhibit HSCs activation	[127]
BMSC	-	Wnt/β-catenin	CCl4-treated rats	IV	reduce liver inflammation, improves liver function, reduce collagen deposition	LX-2	inhibit HSCs activation	[119]
BMSC	miR-26a	-	CCl4-treated mice	IV	alleviate LF	LX-2	promote HSCs ferroptosis	[120]
BMSC	miR-136-5p	GNAS/STAT3	Mice with CLD	IV	reduce liver inflammation, improve liver function,	RAW264.7	reduce liver inflammation, improve liver function	[121]
BMSC	circCDK13	miR-17-5p/KAT2B	Thioacetamide-treated mice	Intraperitoneal injection	reduce collagen deposition and fibrosis in the liver	-	-	[122]
BMSC	circDIDO1	miR-141-3p/PTEN/AKT	-	-	-	LX-2	inhibit HSCs activation	[129]
ADSC	-	PI3K/Akt/mTOR	CCl4-treated mice	IV	recover liver injury, reduce hepatic inflammation	LX-2	arrest cell cycle, inhibit HSCs activation, alleviate LF	[123]
ADSC	miR-223-3p	-	mice with NAFLD	IV	repress lipid accumulation	Hepatocyte	reduce lipid accumulation	[125]
ADSC	miR-150-5p	-	CCl4-treated mice	IV	reduce liver inflammation, alleviate hepatic injury	LX-2	inhibit HSCs activation	[126]
ADSC	-	-	CCl4-treated mice	IV	improve liver function, reduce liver collagen deposition	LX-2	suppresses HSCs activation and proliferation	[124]
HepG2	miR-423-5p	-	CCl4-treated mice	IV	alleviate LF	LX-2	inhibit HSCs activation	[135]
HepG2	-	TGF-β1/Smad, Nrf2/HO-1	CCl4-treated mice	IV	ameliorate oxidative stress and inflammation	LX-2	reduce liver inflammation, inhibit HSCs activation	[136]
HLSC	miR-146a-5p	-	-	-	-	LX-2	inhibit HSCs activation	[138]
HLSC	-	-	mice with NASH	IV	reduce liver inflammation	-	-	[139]
HLSC	miR-142a-5p	miR-142a-5p/CTSB	CCl4-treated mice	IV	alleviate hepatic injury	RAW246.7	affect the polarization of macrophages	[140]
NK Cell	miR-223	-	CCl4-treated mice	IV	alleviate liver damage, reduce collagen deposition	LX-2	inhibit autophagy, inhibit HSCs activation	[141,142]
Tea	miR-44	-	CCl4-treated mice	IV	reduce collagen deposition	LX-2	inhibit HSCs activation	[143]

hucMSC: human umbilical cord MSCs; BMSC: bone marrow MSC; ADSC: adipose MSC; HepG2: Primary Hepatocyte G2; HLSC: human liver stem cells; HSC: hepatic stellate cell; IV: intravenous injection; LX-2: Human hepatic stellate cell line; RAW264.7: Mouse Mononuclear macrophages cells; TH17: helper T cell 17.

## 5. Perspectives and Challenges

Although significant progress has been made in understanding the biology of EVs, their pathogenic role in LF, and their therapeutic potential, several key challenges hinder the translation of EV research from the laboratory to clinical practice. A major obstacle is the lack of standardized protocols and robust quality control measures [166]. First, there is a no globally accepted standard for isolating and purifying EVs. Variations in laboratory conditions and procedures significantly influence the characteristics of the obtained EVs preparations. To date, no single method excels in all aspects, including yield, purity, ease of operation, cost, and vesicle integrity. Current mainstream techniques each have distinct features: ultracentrifugation, particularly density gradient centrifugation, serves as the traditional “gold standard” and achieves relatively high purity, but is time-consuming and can compromise vesicle integrity. Size-exclusion chromatography and tangential flow filtration offer a favorable balance of yield, purity, and integrity but are limited in throughput. Polymer-based precipitation is simple and yields high quantities, but results in lower purity due to co-precipitation of contaminants like lipoproteins. Immunoaffinity capture isolates high-purity subpopulations using specific surface markers (e.g., CD9, CD63), but is costly and provides low yields. Emerging technologies such as microfluidics represent a promising direction toward automation, integration, and high-throughput processing [167,168,169,170,171]. A critical issue is that high- and low-density lipoproteins, abundant in blood, share similar size and density with EVs, constituting a major source of contamination. This overlap can severely compromise the accuracy of downstream proteomic or RNA sequencing analyses and potentially lead to erroneous conclusions. Second, existing technologies struggle to precisely distinguish EV subpopulations derived from different cell origins, a significant limitation given that EVs from different sources may exert disparate or even opposing functions [171]. Furthermore, many mechanistic studies rely on in vitro cell models, but culture conditions can induce cellular stress, altering EV secretion profile and cargo, potentially causing results to deviate from the actual in vivo pathophysiological state. Similarly noteworthy, many studies, upon observing changes in specific nucleic acids or proteins within EVs, infer them to be functional cargo. However, rigorous in vivo gain-or loss-of-function experiments are often lacking to confirm a causal relationship, posing a risk of overinterpretation [172]. The stability of EVs presents another practical challenge. Isolated EVs require appropriate storage conditions to maintain their integrity. Long-term cryopreservation not only increases logistical difficulty and cost but may also impair EV bioactivity [173]. Finally, safety is paramount for clinical applications. studies suggest that EVs may carry risks of immunogenicity, immunotoxicity, and even potential carcinogenic effects. For instance, tumor-derived EVs possess dual potential to either promote or suppress tumorigenesis [174]. Therefore, future research must establish more precise and reproducible isolation and characterization techniques, integrate rigorous in vitro and in vivo functional validation systems, and further confirm the specificity, sensitivity, and safety of EVs as biomarkers or therapeutic agents in large-scale clinical cohorts.

Despite these challenges, EVs hold significant potential for clinical application as therapeutic agents for LF, owing to their inherent biological properties, which can be further enhanced through engineering modifications [175]. For example, transfecting producer cells using lentiviral or adenoviral vectors can generate EVs that overexpress particular antifibrotic factors, thereby enhancing their anti-fibrotic effects on recipient cells. As previously discussed, such engineered EVs represent a powerful and novel delivery platform for treating LF. Nevertheless, these engineering approaches are not without limitations [176]. For instance, genetically modified parental cells may package only a fraction of the intended cargo into EVs, due to inefficient encapsulation and reagent loss. Additionally, the introduction of exogenous cargoes risks off-target effects by competing with or disrupting endogenous cellular components. The immunogenic profile of EVs may also be altered following engineering, a factor that remains insufficiently characterized [177]. Notably, the clinical translation of EV-based therapies faces rigorous regulatory and manufacturing hurdles. As biologically derived products, they must meet stringent criteria for sterility, endotoxin levels, residual host-cell DNA, and batch-to-batch consistency, including vesicle size, surface markers, and cargo composition. While EVs are generally considered to have low immunogenicity, this property cannot be overlooked and may be affected by engineering, necessitating thorough preclinical safety assessment [178]. Therefore, although engineering enhances the therapeutic potential of EVs, it also introduces new variables in toxicity, safety, and immunogenicity that require careful evaluation. The selection of appropriate and efficient modification methods, based on the inherent properties of EVs, is essential to improve targeting and therapeutic efficacy. In summary, engineered EVs offer a promising cell-free therapeutic strategy for liver disorders and warrant continued investigation to realize their full clinical potential.

Moreover, situating engineered EVs within the broader perspective of nanomedicine delivery platforms and comparing them with synthetic nanocarriers like liposomes allow for a more objective assessment of their translational potential. For the delivery of small molecules such as nucleic acids, EVs and lipid nanoparticles (LNPs) represent bio-derived and synthetically engineered strategies, respectively, with characteristics that are both competitive and complementary. Following intravenous administration, both EVs and LNPs are predominantly accumulate in the liver and spleen, though through distinct mechanisms [179]. The biodistribution of LNPs is precisely regulated by their physicochemical properties: a size below 100 nm is generally required to pass through hepatic sinusoid fenestrations for hepatocyte targeting, while surface charge modulation can shift tropism toward the spleen or lungs [180]. In contrast, EV distribution is largely governed by their cellular origin. Specific integrins, tetraspanins, and surface glycan profiles mediate organotropism, for instance, dendritic cell-derived EVs tend to accumulate in the spleen [181]. Evidence regarding their transfection efficacy is mixed. Under local administration or at lower doses, EVs often demonstrate higher protein expression or gene knockdown efficiency than LNPs, accompanied by lower systemic inflammatory responses. This suggests favorable efficiency and tolerability, likely owing to their natural homing ability and efficient cellular uptake [182]. However, under systemic administration and at higher doses, some LNP formulations can produce stronger pharmacological effects. This indicates that the performance of EVs is highly dependent on their cellular source, route of administration, and preparation process, making direct and universal comparisons difficult [183]. The most pronounced gap between the two technologies lies in clinical translation. LNP technology is well established, with clearly defined critical quality attributes, scalable production, and established regulatory pathways. It has not only enabled several approved siRNA/mRNA therapeutics but has also expanded into lung targeting, in vivo gene editing, and even in vivo engineering of CAR-T cells, representing a cutting-edge frontier [184,185,186]. In stark contrast, despite their considerable biological potential, EVs face challenges in scalable manufacturing, batch-to-batch consistency, low loading efficiency, compositional complexity, and incompletely elucidated mechanisms. These limitations have slowed clinical translation, with only a limited number of related trials currently underway [187]. Looking forward, LNPs serve as a validated and “refined tool,” with current efforts focused on engineering solutions to overcome extrahepatic targeting limitations. EVs, on the other hand, constitute an “intricate system” rich in biological information, yet transforming them into stable, controllable “drug products” remains a formidable task. These paths are not mutually exclusive. Insights from EV biology may inspire the design of next-generation biomimetic LNPs, while the development of semi-synthetic hybrid systems or fully synthetic EV mimetics could retain the functional advantages of EVs while overcoming production and quality control challenges. Together, these approaches may advance the development of next-generation precision nucleic acid delivery platforms.

## 6. Conclusions

In conclusion, this review has summarized the fundamental features of EVs and LF, the underlying pathological mechanisms, and the therapeutic prospects of EVs. Accumulating evidence underscores the crucial role of EVs in initiating and advancing LF, where they act as key mediators of cell–cell and tissue–tissue communication. Moreover, EVs are increasingly recognized not only as biomarkers for LF but also as promising cell-free therapeutic agents. Through the convergence of precision medicine and nano technology, engineered EVs have attracted growing interest, providing innovative strategies for the management of liver diseases. As research in this field progresses, EVs are expected to become integral components in the diagnostic and therapeutic landscape of LF and other hepatic diseases in the near future.

## Figures and Tables

**Table 1:** EVs as biomarkers for LF.

Source of EVs	Liver Diseases (Type)	EV Cargo Sequencing (Type)	EV Cargos/Markers	Comparison	Levels	Ref.
plasma platelets, endothelial cells, leukocytes	MASLD	Protein	CD14/CD16	-	↑	[85]
plasma	MASLD	Protein	GLUT1	HC with MASLD	↑	[86]
serum	HCC	Protein	fibulin-4	HC with HCC	↑	[87]
urine	ALD	Protein	CD10, Syntenin-1, TSG101	Non-cirrhotic with cirrhotic	↓/↑	[88]
serum	LF	miRNA	miR-34c, miR-151-3p, miR-483-5p, miR-532-5p, miR-687	HC with LF	↓	[89]
serum	LF	miRNA	miR-214, miR-199a-5p	HC with LF	↓	[90,91]
serum	PSC, PBC	lncRNA	lncRNA H19	HC with PSC, PBC	↑	[68]
serum, plasma	LF	lncRNA	lncRNA-MALAT1	HC with LF	↑	[92]
plasma	HCC	circRNA	circ_0070396	HC with HCC	↑	[93]
plasma	HCC	circRNA	circ-0051443	HC with HCC	↓	[94]
hepatocyte	Viral hepatitis	miRNA	miR-222	HC with Viral hepatitis	↑	[95]
hepatocyte	Viral hepatitis	miRNA	miR-122, miR-146a	HC with Viral hepatitis	↑	[96]
hepatocyte	MASLD	miRNA	miR-1297	HC with MASLD	↑	[41]
hepatocyte	MASLD	miRNA	miR-128-3p	HC with MASLD	↑	[48]
serum	Viral hepatitis	miRNA	miR-19a	HC with Viral hepatitis	↑	[53]
serum	CHD	miRNA	miR-500	HC with CHD	↑	[59]
serum	CHD	miRNA	miR-103-3p	HC with CHD	↑	[60]

HC: healthy control; MASLD: metabolic dysfunction-associated steatotic liver disease; ALD: alcoholic liver disease; PSC: primary sclerosing cholangitis; PBC: primary biliary cholangitis; CHD: chronic hepatic disease; HCC: hepatocellular carcinoma; LF: liver fibrosis; GLUT1: Glucose transporter protein 1; CD14/16/10: Cluster of Differentiation 14/16/10; TSG101: Tumor Susceptibility Gene 101; MALAT1: Metastasis-Associated Lung Adenocarcinoma Transcript 1; miRNA: microRNA; circRNA: Circular RNA; lncRNA: Long Non-coding RNA; ↑: The level of EV Cargos/Markers increases; ↓: The level of EV Cargos/Markers has decreased.

## Data Availability

No datasets were generated or analyzed during the current study.

## Abbreviations

LF	liver fibrosis
EVs	extracellular vesicles
ECM	extracellular matrix
ALD	alcoholic fatty liver disease
MASLD	metabolic dysfunction-associated steatotic liver disease
sEVs	small extracellular vesicles
lEVs	large extracellular vesicles
MSC	mesenchymal stem cell
MSC-EVs	mesenchymal stem cell-derived EVs
LPS	lipopolysaccharide
PBC	primary biliary cholangitis
FFA	free fatty acids
ROS	Reactive oxygen species
PTEN	Phosphatase and Tensin Homolog
HSCs	hepatic stellate cells
α-SMA	α-Smooth Muscle Actin
TGF-β1	transforming growth factor-β1
TNF-α	tumor necrosis factor-α
PDGF	Platelet derived growth factor
PI3K	Phosphatidylinositol 3-Kinase
MASP1	mannan-conjugated lectin serine protease 1
Hep-EVs	hepatocyte-derived EVs
HNF1α	Hepatocyte nuclear factor 1α
DR5	death receptor 5
PPAR-γ	peroxisome proliferator-activated receptor γ
LIMA1	LIM structural domain and actin-binding 1
AKT	Protein Kinase B
MFN2	mitofusin 2
KLF4	Krüppel-like factor 4
NK-EVs	EVs generated from natural killer cells
NK cell	natural killer cell
HSCs-EVs	HSCs-derived EVs
CCN2	Connective tissue growth factor
TE	transient elastography
MRI	magnetic resonance imaging
GLUT1	Glucose transporter protein 1
uEVs	urine EVs
AFP	alpha-fetoprotein
HCC	hepatocellular carcinoma
hucMSC-EVs	EVs produced from human umbilical cord mesenchymal stem cells
TH17	helper T cell 17
BMSC	bone marrow mesenchymal stem cells
BMSC-EVs	BMSC-derived EVs
ADSC	adipose-derived stromal cells
ADSC-EVs	EVs secreted from adipose mesenchymal stem cells
EMT	epithelial–mesenchymal transition
HLSC	human liver stem cells
ATG7	Autophagy-related 7
TEVs	tea-derived EVs
BMP7	gene bone morphogenetic protein 7
FA	forsythoside A
HA	hyaluronic acid
HCV	hepatitis C virus
HBV	hepatitis B virus
SOCS3	suppressor of cytokine signaling 3
STAT3	signal transducer and activator of transcription 3
PCNA	Proliferating Cell Nuclear Antigen
PINK1	PTEN Induced Putative Kinase 1
COL1A1	Collagen Type I Alpha 1 Chain
SOCS1	suppressor of cytokine signaling 1
SIRT1	Sirtuin 1
IL-6	Interleukin-6
TLR4	Toll-like Receptor 4
SHP	small heterodimeric chaperones
TAA	thioacetamide
Lamp2b	Lysosome-associated membrane protein 2b
DSPE-PEG	1,2-distearoyl-sn-glycero-3-phosphoethanolamine-polyethyleneglycol
mEVs	mammary EVs
Chol-EVs	cholangiocyte-derived EVs
CRISPR-Cas9	clustered regularly interspaced short palindromic repeats and associated Cas9 endonuclease
CCL-2/	C-C Motif Chemokine Ligand 2
CCR-2	C-C Motif Chemokine Receptor 2
LSECs	liver sinusoidal endothelial cells
LSECs-EVs	LSECs-derived EVs
SK1	sphingosine kinase 1
CCL4	carbon tetrachloride
MALAT1	Metastasis-Associated Lung Adenocarcinoma Transcript 1
BECN1	Beclin 1
xCT	cystine antiporter light chain
GPX4	glutathione peroxidase 4
SLIT3	Slit Guidance Ligand 3
PSC	primary sclerosing cholangitis
Wnt/β	Wingless/Integrated/β-catenin
SLC7A11	Solute Carrier Family 7 Member 11
KAT2B	Klysine Acetyltransferase 2B
mTOR	Mechanistic Target of Rapamycin
LOXL2	Lysyl Oxidase Like 2
Nrf2	Nuclear factor erythroid 2-related factor 2
KLF6	Krüppel-Like Factor 6
ZEB1	Zinc Finger E Box Binding Homeobox 1
PIK3R3	Phosphoinositide-3 Kinase Regulatory Subunit 3
HepG2-EVs	EVs from the HepG2 cell line
HLSC-EVs	HLSC derived-EVs
CTSB	Cathepsin B
AST	Aspartate Aminotransferase
ALT	Alanine Aminotransferase
HSTP1	Hepatic Stellate cell-Targeting Peptide 1
5HT1D	5-Hydroxytryptamine Receptor 1D
FDA	Food and Drug Administration
PUMA	p53 Upregulated Modulator of Apoptosis
CcnE1	Cyclin E1
KAT5	Klysine Acetyltransferase 5
LUT	luteolin
LNPs	lipid nanoparticles
CAR-T	Chimeric Antigen Receptor T-cell
ESCRT	endosomal sorting complex required for transport
ASGPR1	asialoglycoprotein receptor 1
MHC	major histocompatibility complex
H2AFJ	H2A Histone Family Member J
MAPK	Mitogen-Activated Protein Kinases
STMN1	Stathmin 1
GNAS	Guanine Nucleotide-binding protein
ERK	Extracellular Signal-Regulated Kinase
CD	Cluster of Differentiation
SPARC	secreted protein acidic and cysteine-rich
